# Potential Zoonotic Pathovars of Diarrheagenic *Escherichia coli* Detected in Lambs for Human Consumption from Tierra del Fuego, Argentina

**DOI:** 10.3390/microorganisms9081710

**Published:** 2021-08-11

**Authors:** Ximena Blanco Crivelli, María Paz Bonino, Mariana Soledad Sanin, Juan Facundo Petrina, Vilma Noelia Disalvo, Rosana Massa, Elizabeth Miliwebsky, Armando Navarro, Isabel Chinen, Adriana Bentancor

**Affiliations:** 1 Universidad de Buenos Aires, Facultad de Ciencias Veterinarias, Microbiología, Buenos Aires 1427, Argentina; mpazbonino@fvet.uba.ar (M.P.B.); msanin@fvet.uba.ar (M.S.S.); aben@fvet.uba.ar (A.B.); 2 Departamento de Epidemiología, Ministerio de Salud de Tierra del Fuego, Ushuaia 9410, Tierra del Fuego, Argentina; jpetrina@tierradelfuego.gov.ar; 3 Laboratorio de Diagnóstico de Tierra del Fuego, Tierra del Fuego, Río Grande 9420, Argentina; lab.diagnostico.tdf@gmail.com; 4 Servicio Fisiopatogenia, Departamento Bacteriología, Instituto Nacional de Enfermedades Infecciosas-ANLIS “Dr. Carlos G. Malbrán”, Buenos Aires 1282, Argentina; rmassa@anlis.gob.ar (R.M.); emiliwebsky@anlis.gov.ar (E.M.); ichinen@anlis.gov.ar (I.C.); 5 Departamento de Salud Pública, Facultad de Medicina, Universidad Nacional Autónoma de México, México City CP 04510, Mexico; arnava@unam.mx

**Keywords:** diarrheagenic *Escherichia coli*, lamb, carrier, Argentina

## Abstract

Diarrheagenic *Escherichia coli* (DEC) pathovars impact childhood health. The southern region of Argentina shows the highest incidence of hemolytic uremic syndrome (HUS) in children of the country. The big island of Tierra del Fuego (TDF) in Argentina registered an incidence of five cases/100,000 inhabitants of HUS in 2019. This work aimed to establish the prevalence of STEC, EPEC, and EAEC in lambs slaughtered in abattoirs from TDF as well as to characterize the phenotypes and the genotypes of the isolated pathogens. The prevalence was 26.6% for *stx*+, 5.7% for *eae*+, and 0.27% for *aag*R+/*aai*C+. Twelve STEC isolates were obtained and belonged to the following serotypes: O70:HNT, O81:H21, O81:HNT, O102:H6, O128ab:H2, O174:H8, and O174:HNT. Their genotypic profiles were *stx*_1c_ (2), *stx*_1c_/*ehx*A (3), *stx*_2b_/*ehx*A (1), *stx*_1c_/*stx*_2b_ (2), and *stx*_1c/_*stx*_2_/*ehx*A (4). Six EPEC isolates were obtained and corresponded to five serotypes: O2:H40, O32:H8, O56:H6, O108:H21, and O177:H25. All the EPEC isolates were *bfp*A- and two were *ehxA*+. By X*baI-*PFGE of 17 isolates, two clusters were identified. By antimicrobial susceptibility tests, 8/12 STEC and 5/6 EPEC were resistant to at least one antibiotic. This work provides new data to understand the ecology of DEC in TDF and confirms that ovine are an important carrier of these pathogens in the region.

## 1. Introduction

*Escherichia coli* is a normal inhabitant of human intestinal microbiota and other warm-blooded animals, but through different mechanisms such as plasmid acquisition, pathogenicity islands, bacteriophages, and transposons, *E. coli* incorporated virulence factors and have become important pathogens [1].

The Argentinian surveillance for DEC infectious diseases focuses mainly on STEC infections in children including diarrhea and bloody diarrhea samples. Particularly in the period from 2015 to 2018, over 1047 clinical samples were received at the National Reference Laboratory (NRL), 485 cases were associated with DEC infections: 266 (54.8%) STEC, 107 (22.1%) EAEC, 48 (9.9%) EPEC, 46 (9.5%) EIEC, and 18 (3.7%) ETEC (NRL report for the National System for Health Surveillance).

STEC is a zoonotic microorganism transmitted to humans by the fecal-oral route associated with the consumption of contaminated food and water, and previous reports documented the person-to-person transmission in Argentina [2,3]. This pathogen can cause human infections ranging from asymptomatic carriage to mild or severe diarrhea, hemorrhagic colitis, and hemolytic uremic syndrome (HUS), which can lead to death. HUS is of concern in Argentina because the country reports the highest incidence worldwide, where the children under five years old are the most vulnerable population affected. The National Surveillance System reported, in 2019, 342 HUS cases, 270 of them corresponded to children under five-years-old, with an incidence of 0.76 cases in 100,000 inhabitants [4]. The incidence is not uniform and varies across the country and by seasons with an increase during warm months [5]. In terms of acute diarrhea, in 2019, 124,167 cases were reported with a cumulative incidence of 4306.1 cases per 100,000 inhabitants [4], but their incidence varied across the country, with a high level notified in the southern region of Argentina. Besides STEC, EPEC is another pathovar that impacts public health and causes diarrhea, leading to a range of symptoms in children from mild to severe, and in some cases could be fatal [6,7].

EAEC is a globally important pathogen that causes acute and persistent diarrhea in both children and adults, and can lead to death [8]. In Argentina, EAEC is identified as the second most important pathogen in terms of diarrheas. Furthermore, the emergence of a new pathotype characterized as EAEC O104:H4 with the ability to produce Stx, highly virulent for humans, was described associated with the most important outbreak in Germany, with 3128 cases of acute gastroenteritis mainly in adults, 782 cases of HUS, and 46 deaths [9]. After this outbreak, the protocol for *agg*R and *aai*C (EAEC marker) was incorporated into the workflow for routine diagnosis for DEC and new pathotypes [10], and hybrid strains with similar characteristics have been reported. In 2015, a hybrid EAEC/STEC strain O59:NM[H19] was isolated in Argentina from a child who carried the bacteria for more than 100 days [11], and that serotype with the same virulence profile had been previously isolated in Germany in 2010.

Considering the high incidence of HUS and diarrhea cases in the southern region of the country, the present study was focused on Tierra del Fuego (TDF), where sheep production stands out in the chain from farm-to-plate. Taking into account the frequency of the different categories of DEC in diarrheas and the high association of STEC to HUS cases, the pathovars STEC, EPEC, and EAEC were selected to be investigated in this study.

The big island of TDF is located on the southern archipelago of Argentina and has a surface area of 21,000 km^2^ with 126,998 inhabitants distributed principally in three important cities, Ushuaia (56,956 inhabitants), Tolhuin (3004 inhabitants), and Río Grande (66,938 inhabitants). Because of the characteristics of the area, with steppe and cold semi-desert, the sheep farming established on larges ranches has been an important livestock production in the region due to local consumption and exportation.

Each year, 70,000 heads of ovine are slaughtered, with 90% used for local consumption and only 10% for export. There are two main public abattoirs, one in Ushuaia and the other in Río Grande, which slaughter bovine and ovine, and a third is a private abattoir, also located in Río Grande dedicated only to ovine production, accounting for 70% of ovine slaughters in TDF. Lamb slaughter is mainly concentrated during the period from November to April. The meat and meat products obtained from the municipal abattoirs can only be marketed in the province, and their traditional retail store for fresh meat is through the butcher shop in addition to supermarkets, which gain importance in TDF, by the scale and by the vertical integrations they carry out [12].

Livestock farming for sheep distributed throughout TDF consists of flocks in large land extensions, with a density of one animal per hectare. Besides farming for sheep, many farms also produce cattle on their lands. Due to sanitary restrictions, animals from other parts of Argentina are not allowed to be transported into TDF.

Regarding the antimicrobial susceptibility of the circulating strains, the increase in antimicrobial resistance (AMR) in both human and veterinary medicine has become relevant worldwide. During the 71st General Assembly of the United Nations (2016), AMR was declared one of the main global threats based on health problems and the consequences on trade, production, and transport. In 2015, Argentina started the National Program for the Surveillance of Antimicrobial Resistance in animals destined for human consumption, which has focused on AMR screening in cattle, pigs, and poultry. Because the consumption of sheep does not have a homogeneous distribution in the country, sheep have not been included in this analysis, generating a gap in the role of sheep in the AMR.

As lamb could be a reservoir for DEC with potential impact on human health and their consumption predominates in TDF, this work aimed to establish the prevalence of STEC, EPEC, and EAEC in lambs slaughtered in abattoirs from TDF as well as to characterize the phenotypes and the genotypes of the isolated pathogens.

## 2. Materials and Methods

### 2.1. Samples

A cross-sectional epidemiological study was carried out between February 2017 and April 2018 to determine the prevalence of STEC, EPEC, and EAEC in lambs raised for consumption in TDF. The sampling was carried out at the three abattoirs in TDF during lamb slaughter months (January, February, March, and April).

The sample size was calculated using OpenEpi, version 3.01 software [13], considering a standard normal distribution (Z = 1.96), a precision of 15%, a 99% confidence level, and an estimated prevalence based on previous data of 0.5%. According to that, the sample size was estimated at 74 animals per abattoir.

Only one herd of sheep from a farm entered the slaughterhouse every day. The animals waited in a pen for no more than 24 h until slaughtered. Animals were swabbed at the rectum immediately after being slaughtered, and swabs were maintained in Stuart transport medium (Oxoid, Basingstoke, UK).

An epidemiological log was produced to register sampling date, category (lamb or sheep), production (extensive or intensive), farm of origin, slaughterhouse (ovine or mixed), and slaughterhouse location (Ushuaia, or Río Grande) for each sample.

The study was approved by the Institutional Committee on Animal Care and Use of Experimental Animals (CICUAL; No 2016/45) of the Universidad de Buenos Aires, Faultad de Ciencias Veterinarias.

### 2.2. Detection and Isolation of DEC

Each swab sample was enriched in 5 mL trypticase soy broth (TSB) (Oxoid, Basingstoke, UK) and incubated at 37 °C for 6 h, after that, enrichment broths TSB were streaked onto MacConkey agar (MAC) (Oxoid, Basingstoke, UK) and incubated at 37 °C for 18 h. Then, the culture from the confluence zone was picked up and suspended in 200 μL of sterile ultrapure water in microtubes, placed in a heat block (Labnet, Edison, New Jersey, USA) at 100 °C for 10 min, and centrifuged 1300 rpm for 5 min. The supernatant was used as a DNA template for screening PCR as described below to detect pathovar marker genes (Table S1). Positive plates to marker genes were selected and 50 single colonies with *E*. *coli* morphology were picked up [14] to point-inoculate on MAC, and incubated at 37 °C for 18 h. In the case of being unable to pick up 50 colonies from the positive plate, a loop of the confluence from the positive plate was streaked onto a second MAC plate, which was incubated at 37 °C for 18 h. The 50 colonies were organized in five pools of 10 colonies each, and pools were then subjected to PCR. For pools positive to the marker gene, the single colonies were analyzed individually again. Isolates containing one or more marker gene were streaked onto trypticase soy agar (Oxoid, Basingstoke, UK), confirmed to carry up the selected gene, and finally preserved at −196 °C for further analysis.

The screening of STEC was carried out by conventional multiplex PCR *stx*_1_, *stx*_2_, and *rfb*O_157_ [15] using the ATCC 25,922 strain as a negative control and *E. coli* EDL933 (O157:H7, *stx*_1_/*stx*_2_/*eae*) as a positive control. The PCR mixture was adjusted to a final volume of 50 μL and contained 0.6 μM of each *stx*_1_F and *stx*_1_R primers, 0.12 μM of each primer *stx*_2_F and *stx*_2_R, and *rfb*O_157_F and *rfb*O_157_R, 200 μM of each dNTP (Promega, Madison, Wisconsin, USA), 1.5 mM of MgCl_2_, and 1 U of Taq DNA polymerase (Promega, Madison, Wisconsin, USA), and finally 6 μL of the DNA template. PCR was performed in a Mastercycler Gradient (Eppendorf, Hamburg, Germany).

For EPEC, a single PCR using primers that amplify a 346 bp-fragment of the conserved region of the *eae* gene that encodes for the intimin reported by Blanco et al. (2005) [16] was done using the ATCC 25,922 strain and *E. coli* EDL 933 (O157:H7, *stx_1_/stx_2_/eae*) as the negative and positive control, respectively. The assay conditions were as follows: 50 µL-PCR mixture contained 0.12 µM (each) of the two *eae*-specific primers, 200 µM of each dNTP (Promega, Madison, Wisconsin, USA), 2 mM MgCl_2_ (Invitrogen, Vinius, Lithuania), 1 U of Taq Platinum (Invitrogen, Vinius, Lithuania) DNA polymerase, and 8 μL of the DNA template.

Two single PCRs were done for EAEC, one using primers for the *aai*C gene [17], and the other with primers for *agg*R gene [18]. The ATCC 25,922 strain was used as a negative control and strain HH8 from Statens Serum Institut (*agg*R/*aai*C) was used as a positive control. For *aai*C, the assay conditions were as follows: 50 µL-PCR mixture contained 0.2 µM (each) of the two *aaiC*-specific primers, 200 µM of each dNTP, 2 mM MgCl_2_, 1 U of Taq DNA polymerase (Promega, Madison, Wisconsin, USA), and 10 μL of the DNA template. For *aag*R, the assay conditions were as follows: 50 µL-PCR mixture contained 0.8 µM (each) of the two *aggR*-specific primers, 200 µM of each dNTP (Promega, Madison, Wisconsin, USA), 3 mM MgCl_2_ (Invitrogen, Vinius, Lithuania), 1 U of Taq Platinum DNA polymerase (Invitrogen, Vinius, Lithuania), and 8 μL of the DNA template.

Ten μL of each PCR product obtained was electrophoresed on 2% agarose gels (Promega, Madison, Wisconsin, USA) and stained with ethidium bromide (Promega, Madison, Wisconsin, USA).

In addition, the specific protocol for the detection of STEC O157 was performed as follows. Each sample was enriched in 5 mL de tellurite cefixime (BioMérieux, Marcy-l’Etoile, France) TSB and incubated at 37 °C for 6 h. The screening was based on an immunochromatography test (Reveal^®^ *E. coli* O157) (Neogen, Lansing, Michigan, USA) performed following the supplier’s recommendations. Immunomagnetic separation for O157 (Neogen, Ayr, Scotland) and screening by multiplex PCR *stx*_1_, *stx*_2_, and *rfb*O_157_ genes were carried out for positive samples.

### 2.3. Biochemical Identification and Characterization of DEC

The isolates were phenotypically characterized (morphology, Gram stain, and motility) and were confirmed as *E. coli* through biochemical tests [oxidase (Britania, Buenos Aires, Argentina), catalase (Britania, Buenos Aires, Argentina), OF glucose (Millipore, Darmstadt, Germany), indole production (Millipore, Darmstadt, Germany), mixed-acid-fermentation (methyl red test) (Millipore, Darmstadt, Germany) or butylene glycol (Voges Proskauer) (Millipore, Darmstadt, Germany), Simmons citrate (Oxoid, Basingstoke, UK), and sulfur dioxide production (Millipore, Darmstadt, Germany)] [19]. For STEC, the identification of *stx*_1_ subtypes (*stx*_1a_, *stx*_1c_, *stx*_1d_) and *stx*_2_ subtypes (*stx*_2a_, *stx*_2b_, *stx*_2c_, *stx*_2d_, *stx*_2e_, *stx*_2g_) was carried out by conventional PCR amplification using HotStarTaq Master Mix Kit (Quiagen, Hilden, Germany) [20] (Table S2). The *eae* gene and autoagglutinating adhesin (*saa*) were tested by PCR [16,21] (Table S2). The assay conditions for *saa* PCR were as follows: 50 µL-PCR mixture containing 0.12 µM (each) of the two *saa*-specific primers, 200 µM of each dNTP, 1.5 mM MgCl_2_, 1 U of Taq DNA polymerase (Promega, Madison, Wisconsin, USA), and 4 μL of the DNA template. For *ehx*A gene the assay conditions were as follows: 50 µL-PCR mixture contained 1 µM (each) of the two *ehx*A-specific primers, 200 µM of each dNTP, 2 mM MgCl_2_, 1 U of Taq DNA polymerase (Promega, Madison, Wisconsin, USA), and 6 μL of the DNA template. The EPEC isolates were confirmed by the absence of *stx*_1_ and *stx*_2_ genes. Additionally, the *bfp*A and *ehx*A genes were tested by PCR in all isolates [21,22] (Table S2). For the *bfp*A gene, the assay conditions were as follows: 50 µL-PCR mixture contained 0.2 µM (each) of the two *bfp*A-specific primers, 200 µM of each dNTP, 2 mM MgCl_2_, 1 U of Taq DNA polymerase (Promega, Madison, Wisconsin, USA), and 5 μL of the DNA template.

### 2.4. Serotyping of DEC

The isolates were serotyped by agglutination assays with wild rabbit antisera: 187 sera were used against somatic antigens (O) [23] and 53 sera were used against flagellar antigens (H) [24] at the Facultad de Medicina, Universidad Nacional Autónoma de México (UNAM), México.

To confirm the O174 serogroup, two single PCRs to detect O174*wzx* and O174*wzy* genes [25] were performed in all the O174 isolates [26]. *E. coli* EDL933 strain (O157:H7) and *E. coli* DG131/13 (O174:H8) were used as the negative and positive control, respectively. The 50 µL PCR mixture contained 5 µL of reaction buffer, 0.05 µM (each) of the two specific primers, 200 µM of each dNTP, 1.75 µM MgCl_2_ sin, USA), 1 U of Taq DNA polymerase (Promega, Madison, Wisconsin, USA), and 5 µL of the DNA template.

### 2.5. Antimicrobial Susceptibility Testing

Antimicrobial susceptibility against amoxicillin-clavulanate (2:1, 30 µg), imipenem (10 µg), aztreonam (30 µg), gentamicin (120 µg), nalidixic acid (30 µg), ciprofloxacin (5 µg), streptomycin (10 µg), nitrofurantoin (300 µg), tetracycline (30 µg), chloramphenicol (30 µg), cefotaxime (30 µg), and ceftazidime (30 µg) was evaluated using the Bauer–Kirby methodology in the isolates [Oxoid monodisks (Oxoid, Basingstoke, UK), and Rosco tablets (Rosco, Taastrup, Denmark)] according to the Clinical Laboratory and Standards Institute (CLSI) recommendations. Isolates were classified as being susceptible, showing reduced susceptibility (r), or being resistant (R).

### 2.6. Pulsed-Field Gel Electrophoresis (PFGE)

The macrorestriction fragment separation by PFGE was performed using the 24-h PulseNet standardized PFGE protocol for *E. coli* non O157:H7 (Centers for Disease Control Prevention (2013) with minor modifications. Restriction was carried out with 30 U of *Xba*I (Fermentas, Vilnius, Lithuania). PFGE images of gels were captured using Doc-It 2000 (Bio-Rad, Hercules, California, USA). The TIFF images obtained by PFGE were analyzed with the BioNumerics version 5.1 software package (Applied Maths, Sint-Martens-Latem, Belgium). The relatedness among the patterns was estimated by the proportions of shared bands after applying the Dice coefficient. The UPGMA method was used to generate dendrograms with 1.5% tolerance values. The analysis of the patterns was confirmed visually.

### 2.7. Statistical Analysis

Statistical analysis was carried out using the test of differences between proportions (InfoStat 2016e) [27].

## 3. Results

**Figure 1:**
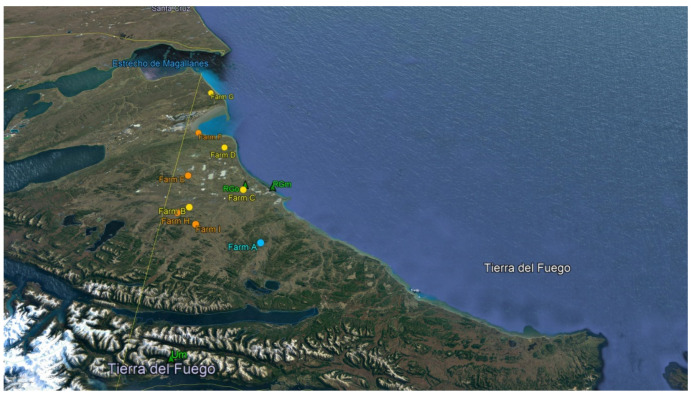
Farms where lambs from this study were raised and slaughterhouses in TDF. Orange dots show farms with STEC (*stx*+/*eae*−) positive animals at the screening, yellow dots show farms with STEC (*stx*+/*eae*−) and EPEC (*stx*−/*eae*+) positive animals at the screening, light blue dot shows farms with STEC (*stx*+/*eae*−), EPEC (*stx*−/*eae*+), and EAEC (*aag*R+/*aai*C+) positive animals at the screening, green triangle show abattoirs.

Between February 2017 and April 2018, 368 swab samples from ovine slaughtered in abattoirs in the TDF province were collected for analysis. All the animals sampled belonged to the lamb category, they were raised on pasture, and did not show signs of disease. Lambs sampled were raised in nine farms (A–I) (Figure 1). A total of 129 samples from the sole ovine-abattoir in Río Grande (RGo) came from farms C (23.26%), D (23.26%), E (22.48%), F (15.5%), and G (15.5%). One hundred and thirty-one samples from the mixed abattoir in Río Grande (RGm) came from farms A (22.9%), B (22.9%), G (13.74%), H (17.56%), and I (22.9%). The 108 samples collected from the mixed abattoir (sheep and cattle) in Ushuaia (Um) came from farm A.

**Figure 2:**
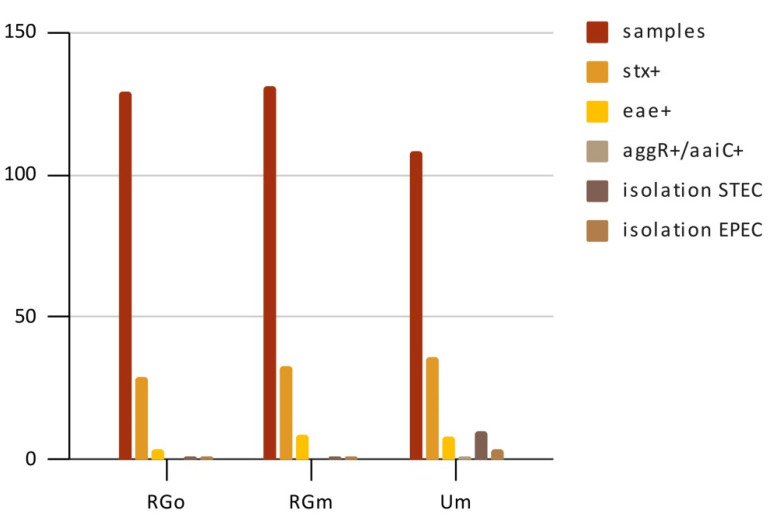
Detection and isolation of STEC, EPEC, and EAEC at abattoirs in TDF. RGo: Rio Grande; slaughterhouse exclusive for sheep. RGm: Rio Grande; slaughterhouse for both cattle and sheep. Um: Ushuaia; slaughterhouse for both cattle and sheep.

Out of 368 swab samples, 98 were positive for *stx* at the screening stage, with a prevalence of *stx*+ of 26.63% (98/368). The distribution regarding the slaughterhouse was 29/129 from RGo, 33/131 from RGm, and 36/108 from Um. A total of 12 STEC isolates out of 98 *stx*+ samples were obtained (1 from RGo, 1 from RGm, and 10 from Um) (Figure 2), their distribution and characterization are shown in Table 1. The STEC isolates belonged to the following serotypes: O70:HNT (1/12), O81:H21 (1/12), O81:HNT (1/12), O102:H6 (1/12), O128ab:H2 (5/12), O174:H8 (2/12), and O174:HNT (1/12), and the genotype observed were *stx*_1c_ (*n* = 2), *stx*_1c_/*ehx*A (*n* = 3), *stx*_2b_/*ehx*A (*n* = 1), *stx*_1c_/*stx*_2b_ (*n* = 2), and *stx*_1c_/*stx*_2b_/*ehx*A (*n* = 4), all of them being *eae*- (Table 1).

According to EPEC detection, 21 swabs were *eae*+ and *stx*- at the screening stage (4/129 from RGo, 9/131 from RGm, and 8/108 from Um) with a prevalence of *eae*+ of 5.7% (21/368). A total of six EPEC isolates were obtained (1 from RGo, 1 from RGm, and 4 from Um) (Table 2). The EPEC isolates belonged to five serotypes: O2:H40 (1/6), O32:H8 (1/6), O56:H6 (1/6), O108:H21 (1/6), and O177:H25 (2/6). All EPEC isolates were *bfp*A-, turning out to be atypical (aEPEC), and only the two O177:H25 EPEC were *ehx*A+. The distribution of EPEC isolations and their characterization are shown in Table 1.

In terms of EAEC screening, only one sample was *agg*R+ and *aai*C+ (0/129 from RGo, 0/131 from RGm, and 1/108 from Um). Although the prevalence of *agg*R+/*aai*C+ was established at 0.27% (1/368), no EAEC was isolated.

Antimicrobial susceptibility tests showed that 8/12 STEC and 5/6 EPEC were resistant or had reduced susceptibility to at least one antibiotic (Table 2).

Test of differences in proportions showed that the detection of STEC (98/368) in slaughtered lambs from TDF was significantly higher than the detection of EPEC (21/368) (difference = 0.20924; Z = 7.71; *p* = 0.000; lower limit = 0.15824, upper limit = 0.26024).

A significant statistical difference could be seen (*p* < 0.05) in the detection of STEC by the farm of origin (farms A [difference = 0.11884; Z = 2.50; *p* = 0.0125, lower limit = 0.02327; upper limit = 0.21441], B [difference: 0.21815; Z = 2.59; *p* = 0.0096, lower limit = 0.03378; upper limit = 0.40252], and D [difference = 0.21815; Z = 2.59; *p* = 0.0096, lower limit = 0.03378; upper limit = 0.40252]), and detection of EPEC by the farm of origin (farm A [difference = 0.08261; Z = 3.31; *p* = 0.0009; lower limit = 0.02674; upper limit = 0.13848]). Indeed, STEC isolates were obtained from lambs that came from farms A, I, and G; and EPEC were obtained from lambs that came from farms A and G.

No significant differences were observed (*p* > 0.05) between slaughterhouse (ovine or mixed), or slaughterhouse location.

**Figure 3:**
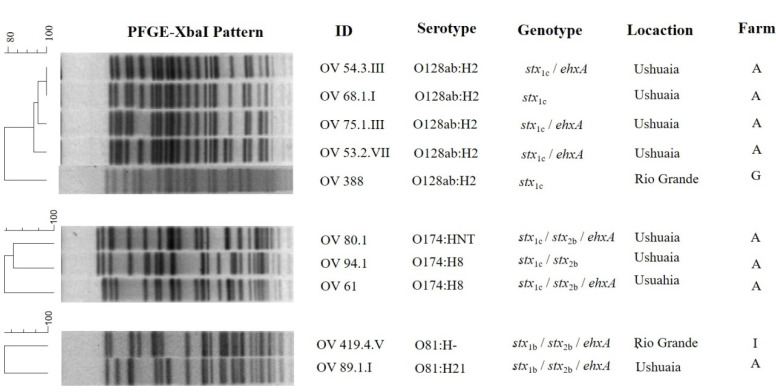
Clonal relationship of STEC obtained from lambs in TDF.

**Figure 4:**
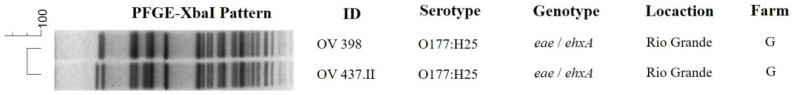
Clonal relationship of EPEC obtained from lambs in TDF.

Out of the total strains, 17 (11 STEC and 6 EPEC) were subtyped by X*baI-*PFGE; just one STEC O70:HNT strain was untypeable. To determine the clonal relationship between the strains of the same serogroup, the PFGE patterns corresponding to STEC strains belonging to O81 (*n* = 2), O174 (*n* = 3), O128 (*n* = 5), and those of EPEC O177 (*n* = 2) were analyzed (Figure 3 and Figure 4). The remaining strains yielded unique *Xba*I-PFGE patterns.

Among the STEC O128 (*n* = 5), two strains that belonged to farm A in Ushuaia were included in cluster I (100% similarity); the other two strains from the same farm shown patterns with >93% similarity with the cluster, with a difference of just 2–3 bands. The remaining strain from farm G in Río Grande was less than 80% similar to the others in STEC O128 mentioned. The STEC O174 and O81 strains were different by the comparison of the *Xba*I-PFGE patterns, with less than 80% similarity, respectively.

The EPEC O177:H25 strains with the same virulence profile were almost identical with 95% similarity (cluster II), and a difference of just two bands. Both were from the same farm G, but processed in different slaughterhouses.

None of the *Xba*I-patterns obtained in the framework of this study showed a close relationship with any human strains included in the National Database.

## 4. Discussion

This study aimed to identify the prevalence of DEC markers in lambs for human consumption in TDF, which could have an impact on children´s health. Previous studies have reported prevalence in ovine, which were observed to be considerably variable between authors. McCarthy et al. (2021) [28] calculated the relative prevalence of STEC in ovine feces from published data, which was established in 33.3% with ranges from 0.9% to 90.0%; according to this analysis, our prevalence results at 26.63% were similar. Despite the fact that we used a specific diagnosis route for STEC O157 detection, which included a selective enrichment, immunocromatography, and IMS, we were unable to detect STEC O157 in contrast to other authors [29,30,31]. These variations can be explained not only in terms of the type of search carried out in each laboratory, but also the animal category, the number of samples, and sampling method.

A total of 98 samples were *stx*+ at the screening and only 12 STEC isolates were obtained, although 50 colonies were picked up from each positive agar plate. The fact that the STEC isolation is from rectal swabs makes it difficult as they are considered microbiologically complex and STEC also could be in low load, making its isolation more difficult. Furthermore, non-O157 serogroups have no biochemical characteristic to distinguish STEC from those commensal *E. coli* strains. Therefore, STEC isolation could be labor intensive, which means to direct the analysis focused in virulence genes by PCR to identify the positive colonies on agar plates. Although there are methodologies to increase the probability of isolation such as the immunomagnetic separation prior to PCR, this technique has only been developed for few serogroups mainly related to *eae*+, which cause severe diseases in human. Indeed, different authors use different concepts for analysis to get the prevalence; this could be calculated based on marker gene detection without isolation [32,33] or using the number of isolates [29,30,31]. To add to the gap in recognizing colonies carrying marker genes detected, we assumed the use of marker gene prevalence. Due to the difficulties to isolate positive colonies and that the PCR is the gold standard test to detect these pathogens, we consider the screening result to calculate the prevalence.

Among the non-O157 serogroups isolated in our study, the most frequent was O128. The high frequency of this serogroup in ovine samples concurs with previously published data [31,34,35,36]. The serotype O128ab:H2, frequently isolated from lambs in TDF in this study, was also detected from HUS cases with or without bacteremia [37], and from patients with diarrhea and meat samples [38]. In Argentina, the O128 serogroup was reported from the NRL related to four asymptomatic individual contacts (O128:NM and O128:H2) of different HUS cases, and also to one animal. On the other hand, the O174 serogroup was isolated from ruminant samples from mixed abattoirs (ovine and bovine) [31,35] and has also been associated with HUS [39]. Moreover, it was recognized as an emerging serogroup associated with severe disease, being the first STEC *eae*-negative serogroup (O174:[H8]; O174:[H21]; O174:[H28]; O174:[HNT]) in terms of prevalence in Argentina. The registers from the 2015–2018 period show that there have been three cases of HUS associated with this serotype as well as six diarrhea cases, three bloody diarrhea cases, and one case of sepsis. In addition, it was isolated from three asymptomatic individuals and four outbreak contacts.

With regard to serotypes O102:H6, O81:H21, and O81:HNT, the first one was isolated in Argentina from a patient with mild and bloody diarrhea [40] and STEC O81:HNT was previously described in Tasmania isolated from raw pork [41], and from cattle in Australia (O81:H21) [42].

Although at the screening stage the coexistence of the *stx* and *eae* genes was detected in three swabs, we could not obtained the isolates; so, it was impossible to distinguish between whether both marker genes were present in a single STEC strain or if two pathovars STEC and EPEC were present simultaneously. Although the presence of *saa* could not be detected in the isolates of this study, other adhesins cannot be ruled out. Recently, a new pathogenicity island called the Locus of Adhesion and Autoaggregation (LAA) [43] was identified and described as an important virulence factor for STEC LEE-negative strains. According to this, it would be interesting to further evaluate the presence of LAA in these isolates.

In our study, *stx*_1c_ and *stx*_1c_/*stx*_2b_ were the main *stx* genotypic profiles shown by the STEC isolates. On the other hand, Vettorato et al. (2009) [44] published s*tx*_1c_/*stx*_2d_ as the predominant profile in STEC obtained from healthy ovine; a different result from this study. However, *stx*_1c_ has been described as a frequent subtype in ovine [38].

In reference to *stx*_2_ subtype, Martins et al. (2015) [35] examined the main *stx*_2_ subtype isolated from sheep, which was *stx*_2b_ alone or with *stx*_2c_ followed by subtypes *stx*_2d_ and *stx*_2a_. As differences in *stx* subtypes were reported between young and old cattle, it is possible that sheep microbiota also shifts according to the age of the animal.

Ghanbarpour et al. (2017) [45] reported aEPEC prevalence as 4.5% in sick lambs, while the prevalence in our study was 5.7% in healthy animals. All the EPEC isolates from our study were aEPEC. According to Nakazato et al. (2004) [46], the ovine could be ecological or epidemiological reservoirs of these aEPEC strains; furthermore, the O2:H40 aEPEC strain was previously identified in ovine and pigs [47]. Other strains of interest include EPEC O56:H6, which was previously isolated from humans with gastrointestinal disorders [48], and aEPEC O108:H21, which was isolated by our working group from *Rattus norvegicus* samples in Buenos Aires, Argentina [49]. As far as we know, there are no previous records of O32:H8 and O177:H25 as aEPEC serotypes. However, O177:H25 has been described as a highly virulent STEC serotype encoding *stx*_2c_ and with similar characteristics to STEC O157 [50]. Considering that the isolated EPEC O177:H25 encoded *ehx*A, it is necessary to consider the possible loss of *stx*-phage. It could be interesting to confirm this hypothesis by a further study to identify the phage insertion site in the strain genome.

A total of 8/12 isolated STEC and 2/6 EPEC exhibited the *ehx*A gene, which encodes for enterohemolysin and is frequently associated with highly pathogenic strains; this fact is in line with Orden et al. (2003) [36], who isolated a large percentage of lamb strains with this characteristic.

In this work, EAEC was detected at the screening stage in one of the lamb samples analyzed. This result differs from those of Wani et al. (2013) [51] who did not detect any EAEC in the lamb samples. This particular DEC has been isolated from other species such as calves, piglets, horses, dogs, and cats [52]. The presentation of this pathovar varies between animal species, and it has been associated with diarrheal disease in swine and cattle [53], and asymptomatic carriage in dogs and cats [54]. The absence of diarrhea in animals suggests that these animals could act as reservoirs for human infection [54]. The screening of EAEC in the positive sample was aagR+/aaiC+, the absence of isolation does not allow us to determine if there is a strain with both genes or more than one strain, the presence of *aai*C+ alone has been previously documented in strains belonging from animals [52]. Moreover, EAEC with both markers *agg*R+/aaiC+ is usually associated with humans, but not with animals.

Fourteen isolates were resistant or showed intermediate sensitivity to at least one antibiotic. The spread of antimicrobial resistance in isolated bacteria from animal food could be a consequence of the inappropriate use of antibiotics, both as prescription drugs and as growth promoters. STEC with resistance profiles to beta-lactams were detected including third-generation cephalosporins (C3G) such as cefotaxime (CTX) and ceftazidime (CAZ). These high-risk strains, where the virulence of the pathovar adds R genes, make it possible to consider the dispersal of resistant DEC genotypes in the community, which can reach the consumer through food. STEC strains with beta-lactam resistance profiles were described by Oporto et al. (2019) [55], but the authors did not use the same panel of antibiotics as we did, therefore not all results can be compared. Our data represent a first contribution to the surveillance of antimicrobial resistance in lambs destined for human consumption in Argentina.

A significant statistical difference could be seen between the detection of the pathogen (STEC or EPEC) by the farm of origin. This could be due to differences in the size of the flocks or the rearing conditions and even to the presence of cattle coexisting on the same farm. Although X*baI*-PFGE showed there is not a great dispersion of clones in the area, the internal movement of animals between farms that would contribute to the spread of strains in the region cannot be ruled out.

Through the comparison of the X*baI-*PFGE patterns of the strains corresponding to the same serogroups O81, O128, O174, and O177, only two clusters were identified. Cluster #1 grouped two strains of O128ab:H2 obtained from the same farm A. Cluster #2 grouped two strains of O177:H25 obtained from animals from the same farm, but slaughtered at a different abattoir in Río Grande.

In conclusion, this study provides initial data to demonstrate that DEC is carried by ovine in TDF, and contributes to our understanding of the epidemiology of these pathogens in the region. STEC carriers were found in all the farms evaluated and EPEC carriers were also detected in 50% of them. In addition, the presence of EAEC was detected in one animal whose farm of origin was positive to the other pathovars evaluated. The results obtained showed the diversity of the strains present in lambs from TDF. STEC (11/12) and EPEC (6/6) recovered in this study were mainly from lambs from farms A and G. Finally, through X*baI-*PFGE, we could confirm the association observed by the analysis of the virulence profile and the demographic information.

Although the southern region of Argentina presents a high incidence of HUS and diarrhea, the presence of DEC had not been studied in ovine herds previously, making this the first prevalence report of DEC in lambs for consumption. As there is a high lamb consumption in TDF, the risk associated should not be ruled out in the evaluation of the farm–to-table process. The next step is to determine DEC levels in the ovine farm-to-table chain. Additionally, the screening of other sources of infection and reservoirs is needed to establish the epidemiology of these pathogens in the region.

## Figures and Tables

**Table 1:** Virulence profiles of DEC isolates from lambs at abattoirs in TDF.

Serotype	*n*	*stx*_1_	*stx*_2_	*rfb*O_157_	*eae*	*saa*	*bfp*A	*ehx*A	Pathovar	Farm	Slaughterhouse	Lamb ID
O2:H40	1	−	−	−	+		−	−	EPEC	A	Um	OV38
O32:H8	1	−	−	−	+		−	−	EPEC	A	Um	OV5
O56:H6	1	−	−	−	+		−	−	EPEC	A	Um	OV52
O70:HNT	1	*stx*_1c_	*stx*_2b_	−	−	−		−	STEC	A	Um	OV91
O81:HNT	1	*stx*_1c_	*stx*_2b_	−	−	−		+	STEC	I	RGm	OV419
O81:H21	1	*stx*_1c_	*stx*_2b_	−	−	−		+	STEC	A	Um	OV89
O102:H6	1	*stx*_1c_	*stx*_2b_	−	−	−		+	STEC	A	Um	OV81
O108:H21	1	−	−	−	+	−	−	−	EPEC	A	Um	OV36
O128ab:H2	3	*stx*_1c_	−	−	−	−		+	STEC	A	Um	OV53/OV54/OV75
−	1	*stx*_1c_	−	−	−	−		−	STEC	A	Um	OV68
−	1	*stx*_1c_	−	−	−	−		−	STEC	G	RGo	OV388
−	1	*stx*_1c_	*stx*_2b_	−	−	−		+	STEC	A	Um	OV61
O174:H8	1	*stx*_1c_	*stx*_2b_	−	−	−		−	STEC	A	Um	OV94
O174:HNT	1	−	*stx*_2b_	−	−	−		+	STEC	A	Um	OV80
O177:H25	1	−	−	−	+	−	−	+	EPEC	G	RGo	OV398
O177:H25	1	−	−	−	+	−	−	+	EPEC	G	RGm	OV437
Total	18	11	7	−	6	−		10	−	−	−	−

−: absence of the gene marker; +: presence of the gene marker.

**Table 2:** Antibiotic susceptibility of DEC from lambs in TDF abattoirs.

Serotype	Pathovar	AMC	S	CTX	AZT	CAZ
O32:H8	aEPEC	s	s	R	s	s
O56:H6	aEPEC	s	r	s	s	s
O70:HNT	STEC	s	r	s	s	s
O81:HNT	STEC	R	s	s	s	s
O81:H21	STEC	s	r	R	r	s
O108:H21	aEPEC	r	s	s	s	s
O128ab:H2	STEC	r	s	s	s	s
	STEC	R	r	s	s	s
	STEC	r	s	s	s	s
	STEC	r	s	s	s	s
O174:H8	STEC	s	R	s	s	R
O177:H25	aEPEC	r	s	s	s	s
	aEPEC	r	s	r	s	s

r: intermediate susceptibility; R: resistant; s: susceptible; AMC: amoxicillin/clavulanic acid; S: streptomycine; CTX: cefotaxime; AZT: aztreonam; CAZ: ceftazidime.

## Data Availability

Not applicable.

## Supplementary Materials

The following are available online at https://www.mdpi.com/article/10.3390/microorganisms9081710/s1, Table S1: Oligonucleotides used for screening and isolation of DEC pathovars, Table S2: Oligonucleotides used for characterization of DEC isolates.

## References

[1] Williams N.D., Torres A.G., Lloyd S.J., Torres A.G. (2010). Evolution and epidemiology of diarrheagenic *Escherichia coli*. Pathogenic Escherichia coli in Latin America.

[2] Miliwebsky E., Deza N., Chinen I., Martinez Espinosa E., Gomez D., Pedroni E., Caprile L., Bashckier A., Manfredi E., Leotta G. (2007). Prolonged fecal shedding of Shiga toxin-producing *Escherichia coli* among children attending day-care centers. Rev. Argent. Microbiol..

[3] Zota C.M., Chinen I., Lavayén S., Cepeda M., Deza N., Morvay L., Carbonari C., Rearte A., Rivas M. (2015). Portación de *Escherichia coli* en convivientes de casos de síndrome urémico hemolítico. Salud(i)Ciencia.

[4] Ministerio de Salud Argentina BIV 2021, N559, in press. https://bancos.salud.gob.ar/bancos/materiales-para-equipos-de-salud/soporte/boletines-epidemiologicos.

[5] Rivero M.A., Paccussi J.A., Rodríguez E.M., Parma A.E. (2011). Seasonal variation of HUS occurrence and VTEC infection in children with acute diarrhea from Argentina. Eur. J. Clin. Microbiol. Infect. Dis..

[6] Trabulsi L.R., Keller R., Gomez T.A.T. (2002). Typical and atypical enteropathogenic *Escherichia coli*. Emerg. Infect. Dis..

[7] Torres A.G. (2017). *Escherichia coli* in Latin America—A One Health multidisciplinary approach. Pathog. Dis..

[8] Waldir P.E., Navarro-García F., Torres A.G. (2016). Enteroaggregative *Escherichia coli* (EAEC). Escherichia coli in the Americas.

[9] Navarro-García F. (2014). *Escherichia coli* O104:H4 pathogenesis: An Enteroaggregative *E. coli*/Shiga Toxin-Producing *E. coli* explosive cocktail of high virulence. Microbiol. Spectr..

[10] Miliwebsky E., Deza N., Zolezzi G., Baschkier A., Carbonari C.C., Manfredi E., D’Astek B.A., Chinen I., Rivas M. (2019). Manual de Procedimientos: Escherichia coli Productor de Toxina Shiga en el Marco de la Detección de E.coli diarreigénico. http://sgc.anlis.gob.ar/handle/123456789/2307.

[11] Carbonari C.C., Ricciardi M., Rodríguez Calvo A., Montes A., Deza N.L., Conde Valentino M.A., Zolezzi G., Baschkier A., Vago M., Acosta D. (2019). An Stx-EAEC O59:NM[H19] strain isolated from an hemolytic uremic syndrome case in Argentina. Rev. Argent. Microbiol..

[12] Consejo Federal de Inversiones Cadena de Valor de la Carne Bovina en la Provincial de Tierra de Fuego. http://biblioteca.cfi.org.ar/wp-content/uploads/sites/2/2016/04/item-d_-tdf.pdf.

[13] Dean A.G., Sullivan K.M., Soe M.M. OpenEpi: Open Source Epidemiologic Statistics for Public Health, Version. www.OpenEpi.com.

[14] Ballem A., Gonc¸alves S., Garcia-Meniño I., Flament-Simon S.C., Blanco J.E., Fernandes C., Saavedra M.J., Pinto C., Oliveira H., Blanco J. (2020). Prevalence and serotypes of Shiga toxinproducing *Escherichia coli* (STEC) in dairy cattle from Northern Portugal. PLoS ONE.

[15] Leotta G., Chinen I., Epszteyn S., Miliwebsky E., Melamed I.C., Motter M., Ferrer M., Marey E., Rivas M. (2005). Validación de una técnica de PCR múltiple para la detección de *Escherichia coli* productor de toxina Shiga. Rev. Argent. Microbiol..

[16] Blanco M., Schumacher S., Tasara T., Zweifel C., Blanco J.E., Dahbi G., Blanco J., Stephan R. (2005). Serotypes, intimin variants and other virulence factors of *eae* positive *Escherichia coli* strains isolated from healthy cattle in Switzerland. Identification of a new intimin variant gene (*eae*-2). BMC Microbiol..

[17] Boisen N., Scheutz F., Rasko D.A., Redman J.C., Persson S., Simon J., Kotloff K.L., Levine M.M., Sow S., Tamboura B. (2012). Genomic characterization of enteroaggregative *Escherichia coli* of children in Mali. J. Infect. Dis..

[18] Wieler L.H., Semmler T., Eichhorn I., Antao E.M., Kinnemann B., Geue L., Karch H., Guenther S., Bethe A. (2011). No evidence of the Shiga toxin-producing *E. coli* O104:H4 outbreak strain or enteroaggregative *E. coli* (EAEC) found in cattle faeces in northern Germany, the hostpot of the 2011 HUS outbreak area. Gut Pathog..

[19] MacFaddin J.F. (2003). Pruebas Bioquímicas Para la Identificación de Bacterias de Importancia Clínica.

[20] Scheutz F., Teel L.D., Beutin L., Pierard D., Buvens G., Karch H., Mellmann A., Caprioli A., Tozzoli R., Morabito S. (2012). Multicenter evaluation of a sequence-based protocol for subtyping Shiga toxins and standardizing Stx nomenclature. J. Clin. Microbiol..

[21] Paton A.W., Paton J.C. (2002). Direct detection and characterization of Shiga toxigenic *Escherichia coli* by multiplex PCR for *stx*_1_, *stx*
_2_, *eae*, *ehxA*, and *saa*. J. Clin. Microbiol..

[22] Gunzburg S.T., Tornieporth N.G., Riley L.W. (1995). Identification of enteropathogenic *Escherichia coli* by PCR-based detection of thebundle—forming pilus gene. J. Clin. Microbiol..

[23] Edwards P.R., Ewing W.H. (1986). Edwards and Ewing’s Identification of Enterobacteriaceae.

[24] Orskov F., Orskov I., Bergan T. (1984). Serotyping of *Escherichia coli*. Methods in Microbiology.

[25] Beutin L., Kong K., Feng L., Wang Q., Krause G., Leomil L., Jin Q., Wang L. (2005). Development of PCR assays targeting the genes involved in synthesis and assembly of the new *Escherichia coli* O174 and O177 O antigens. J. Clin. Microbiol..

[26] Cundon C., Marey E., Roldán F., Canosa Montero C.S., Navarro A., Gadea P., Blanco Crivelli X., Babich J., Rocchi D., Kiernicki M.C. (2015). Preliminary detection and characterization of *Escherichia coli* O174 Shiga toxin-producing. Senasa.

[27] Di Rienzo J.A., Casanoves F., Balzarini M.G., González L., Tablada M., Robledo C.W., Infostat Version 2016e Grupo Infostat. 2016; Universidad Nacional de Córdoba, Argentina. https://www.infostat.com.ar/.

[28] McCarthy S.C., Burgess C.M., Fanning S., Duffy G. (2021). An overview of Shiga-toxin producing *Escherichia coli* carriage and prevalence in the ovine meat production chain. Foodborne Pathg. Dis..

[29] Batisti A., Lovari S., Franco A., Di Egidio A., Tozzoli R., Caprioli A., Morabito S. (2006). Prevalence of *Escherichia coli* O157 in lambs at slaughter in Rome, central Italy. Epidemiol. Infect..

[30] Kudva I.T., Hatfield P.G., Hovde C.J. (1996). *Escherichia coli* O157:H7 in microbial flora of sheep. J. Clin. Microbiol..

[31] Rey J., Blanco J.E., Blanco M., Mora A., Dahbi G., Alonso J.M., Hermoso M., Hermoso J., Alonso M.P., Usera M.A. (2003). Serotypes, phage types and virulence genes of Shiga-producing *Escherichia coli* isolated from sheep in Spain. Vet. Microbiol..

[32] Thomas R.R., Brooks H.J.L., O’Brien R. (2017). Prevalence of Shiga toxin-producing and enteropathogenic *Escherichia coli* marker genes in diarrhoeic stools in a New Zealand catchment area. J. Clin. Pathol..

[33] Baker C.A., De J., Bertoldi B., Dunn L., Chapin T., Jay-Russell M., Danyluk M.D., Schneider K.R. (2019). Prevalence and concentration of *stx*+ *E. coli* and *E. coli* O157 in bovine manure from Florida farms. PLoS ONE.

[34] Beutin L., Geier D., Steinrück H., Zimmermann S., Scheutz F. (1993). Prevalence and some properties of Verotoxin (Shiga-like toxin) producing *Escherichia coli* in seven different species of healthly domestic animals. J. Clin. Microbiol..

[35] Martins F.H., Guth B.E.C., Piazza R.M., Cardoso Leao S., Ludovico A., Ludovico M.S., Dahbi G., Marzoa J., Mora A., Blanco J. (2015). Diversity of Shiga toxin-producing *Escherichia coli* in sheep flocks of Paraná State, southern Brazil. Vet. Microbiol..

[36] Orden J.A., Ruiz-Santa-Quiteria J.A., Blanco M., Blanco J.E., Mora A., Cid D., González E.A., Blanco J., De la Fuente R. (2003). Prevalence and characterization of Vero cytotoxin-producing *Escherichia coli* isolated from diarrhoeic and healthy sheep and goats. Epidemiol. Infect..

[37] Buvens G., De Rauw K., Roisin S., Vanfraechem G., Denis O., Jacobs F., Scheutz F., Pierard D. (2013). Verocytotoxin-Producing *Escherichia coli* O128ab:H2 bacteremia in a 27-year-old male with hemolytic-uremic syndrome. J. Clin. Microbiol..

[38] Brett K.N., Ramachandran V., Hornitzky M.A., Bettelheim K.A., Walker M.J., Djordjevic S.P. (2003). *stx*_1c_ is the most common Shiga toxin 1 subtype among Shiga toxin-producing *Escherichia coli* isolated from sheep but not among isolates from cattles. J. Clin. Microbiol..

[39] Rivas M., Miliwebsky E., Chinen I., Roldán C.D., Balbi L., García B., Fiorilli G., Sosa-Estani S., Kincaid J., Rangel J. (2006). Characterization and epidemiologic subtyping of Shiga toxin-producing *Escherichia coli* strains isolated from hemolytic uremic syndrome and diarrhea cases in Argentina. Foodborne Pathog. Dis..

[40] Oderiz S., Leotta G.A., Galli L. (2018). Detección y caracterización de *Escherichia coli* productor de toxina Shiga en niños atendidos en un hospital pediátrico interzonal de la ciudad de La Plata. Rev. Argent. Microbiol..

[41] Manandhar R., Bettiol S.S., Bettelheim K.A., Goldsmid J.M. (1997). Isolation of verotoxigenic *Escherichia coli* from the Tasmanian environment. Comp. Immunol Microbiol Infect. Dis..

[42] Hornitzky M.A., Vanselow B.A., Walker K., Bettelheim K.A., Corney B., Gill P., Bailey G., Djordjevic P. (2002). Virulence properties and serotypes of Shiga toxin-producing *Escherichia coli* from healthy Australian cattle. Appl. Environ. Microbiol..

[43] Montero D.A., Velasco J., Del Canto F., Puente J.L., Padola N.L., Rasko D.A., Farfán M., Salazar J.C., Vidal R. (2017). Locus of adhesion and autoaggregation (LAA), a pathogenicity island present in emerging Shiga Toxin-producing *Escherichia coli* strains. Sci. Rep..

[44] Vettorato M.P., de Castro A.F.P., Cergole-Novella M.C., Camargo F.L.L., Irino K., Guth B.E.C. (2009). Shiga toxin-producing *Escherichia coli* and atypical enteropathogenic *Escherichia coli* strains isolated from healthy sheep of different populations in Sao Paulo, Brazil. Lett. Appl. Microbiol..

[45] Ghanbarpour R., Askari N., Ghorbanpour M., Tahamtan Y., Mashayekhi K., Afsharipour N., Darijani N. (2017). Genotypic analysis of virulence genes and antimicrobial profile of diarrheagenic *Escherichia coli* isolated from diseased lambs in Iran. Trop. Anim. Health Prod..

[46] Nakazato G., Gyles C., Ziebell K., Keller R., Trabulsi L.R., Gomes T.A.T., Irino K., Da Silveira W.D., De Castro A.F.P. (2004). Attaching and effacing *Escherichia coli* isolated from dogs in Brazil: Characteristics and serotypic relationship to human enteropathogenic *E. coli* (EPEC). Vet. Microbiol..

[47] Fröhlicher E., Krause G., Zweifel C., Beutin L., Stephan R. (2008). Characterization of attaching and effacing *Escherichia coli* (AEEC) isolated from pigs and sheep. BMC Microbiol..

[48] Blanco M., Blanco J.E., Dahbi G., Alonso M.P., Mora A., Coira M.A., Madrid C., Juárez A., Bernárdez M.I., González E.A. (2006). Identification of two new intimin types in atypical enteropathogenic *Escherichia coli*. Int. Microbiol..

[49] Blanco Crivelli X., Bonino M.P., Von Wernich Castillo P., Navarro A., Degregorio O., Bentancor A. (2018). Detection and characterization of enteropathogenic and Shiga toxin-producing *Escherichia coli* strains in *Rattus* spp. from Buenos Aires. Front. Microbiol..

[50] Sheng H., Duan M., Hunter S.S., Minnich S.A., Settles M.L., New D.D., Chase J.R., Fagnan M.W., Hovde C.J. (2018). High-Quality complete genome sequences of three bovine Shiga Toxin-Producing *Escherichia coli* O177:H- (*fliC*_H25_) isolates harboring virulent *stx*2 and multiple plasmids. Genome Announc..

[51] Wani S.A., Hussai I., Beg S.A., Rather M.A., Kabli Z.A., Mir M.A., Nishikawa Y. (2013). Diarrhoeagenic *Escherichia coli* and salmonellae in calves and lambs in Kashmir: Absence, prevalence and antibiogram. Rev. Sci. Tech..

[52] Uber A.P., Trabulsi L.R., Irino K., Beutin L., Ghilardi A.C., Gomes T.A., Liberatone A.M.A., de Castro A.F., Elias W.P. (2006). Enteroaggregative *Escherichia coli* from humans and animals differ in major phenotypical traits and virulence genes. FEMS Microbiol. Lett..

[53] Dubreil J.D. (2019). EAST1 toxin: An enigmatic molecule associated with sporadic episodes of diarrhea in humans and animals. J. Microbiol..

[54] Puño-Sarmiento J., Medeiros L., Chiconi C., Martins F., Pelayo J., Rocha S., Blanco J., Blanco M., Zanutto M., Kobayaski R. (2013). Detection of diarrheagenic Escherichia coli strains isolated from dogs and cats in Brazil. Vet. Microbiol..

[55] Oporto B., Ocejo M., Alkorta M., Marimón J.M., Montes M., Hurtado A. (2019). Zoonotic approach to Shiga toxin-producing *Escherichia coli*: Integrated analysis of virulence and antimicrobial resistance in ruminants and humans. Epidemiol. Infect..

