# Supplementary material for: Potential Zoonotic Pathovars of Diarrheagenic Escherichia coli Detected in Lambs for Human Consumption from Tierra del Fuego, Argentina
Source: Microorganisms. 2021 Aug 11;9(8):1710. doi: 10.3390/microorganisms9081710 (PMC8401499; doi:10.3390/microorganisms9081710)
Supplement: Supplementary file 1 [file microorganisms-09-01710-s001.zip › microorganisms-1294260-SI.pdf]

Table S1. Oligonucleotides used for screening and isolation of DEC pathovars

Target gene	Primer sequence	Amplicon	Reference
stx$_1$	GAAGAGTCCGTGGGATTACG	130	Leotta *et al.*,
	AGCGATGCAGCTATTAATAA		2005.
stx$_2$	TTAACCACACCCCACCGGGCAGT	346	
	GCTCTGGATGCATCTCTGGT		
*rfb*O$_{157}$	CGGACATCCATGTGATATGG	259	
	TTGCCTATGTACAGCTAATCC		
eae	GGAACGGCA GAGGTTAATCTGCAG	346	Blanco *et al.*,
	GGCGCTCATCA TAGTCTTTC		2003.
*aai*C	TGGTGACTACTTTGATGGACATTGT	313	Boisen *et al.*,
	GACACTCTCTTCTGGGGTAAACGA		2012.
*agg*R	GTATACACAAAAGAAGGAAGC	254	Wieler *et al.*,
	ACAGAATCGTCAGCATCAGC		2011.

Table S2. Oligonucleotides used for characterization of DEC isolates

Target gene	Primer sequence	Amplicon	Reference
saa	CGTGATGAACAGGCTATTGC	119	Paton and
	ATGGACATGCCTGTGGCAAC		Paton, 2002.
*ehx*A	GCATCATCAAGCGTACGTTCC	534	Paton and
	AATGAGCCAAGCTGGTTAAGCT		Paton, 2002.
bfpA	AAT GGTGCTTGCGCTTGCTGC	326	Gunzburg *et*
	GCCGCTTTATCCAACCTGGTA		*al.*, 1995.
O174*wzx*	TCTAGGACCTGTAGAACA	656	Beutin *et al.*,
	GTAGTTGATCTGAGCGAT		2005.
O174*wzy*	TATGGGTCCTATTACTTTC	759	
	GTATCGGAGATCATTATTAC		
stx$_{1a}$	CCTTTCCAGGTACAACAGCGGTT	478	Scheutz *et al.*,
	GGAAACTCATCAGATGCCATTCTGG		2012.
stx$_{1c}$	CCTTTCCTGGTACAACTGCGGTT	252	
	CAAGTGTTGTACGAAATCCCCTCTGA		
stx$_{1d}$	CAGTTAATGCGATTGCTAAGGAGTTTACC	203	
	CTCTTCCTCTGGTTCTAACCCCATGATA		
stx$_{2a}$	GCGATACTGRGBACTGTGGCC	349 - 347	
	CCGKCAACCTTCACTGTAAATGTG		
	GCCACCTTCACTGTGAATGTG		
stx$_{2b}$	AAATATGAAGAAGATATTTGTAGCGGC	251	
	CAGCAAATCCTGAACCTGACG		
stx$_{2c}$	GAAAGTCACAGTTTTTATATACAACGGGTA	177	
	CCGGCCACYTTTACTGTGAATGTA		
stx$_{2d}$	AAARTCACAGTCTTTATATACAACGGGTG	179 – 280	
	TTYCCGGCCACTTTTACTGTG		
	TCAACCGAGCACTTTGCAGTAG		
	GCCTGATGCACAGGTACTGGAC		
stx$_{2e}$	CGGAGTATCGGGGAGAGGC	411	
	CTTCCTGACACCTTCACAGTAAAGGT		
stx$_{2f}$	TGGGCGTCATTCACTGGTTG	424	
	TAATGGCCGCCCTGTCTCC		
stx$_{2g}$	CACCGGGTAGTTATATTTCTGTGGATATC	573	
	GATGGCAATTCAGAATAACCGCT		
